# Comparative Study of *Moringa* *oleifera* and *Sclerocarya birrea* Extracts in Forensic Urine Analysis

**DOI:** 10.1002/open.70235

**Published:** 2026-07-13

**Authors:** Nare Leah Mojela, Eswaran Prabakaran, Kriveshini Pillay, Phogothi Patrick Komane

**Affiliations:** ^1^ Department of Chemical Sciences Faculty of Science University of Johannesburg Johannesburg South Africa

**Keywords:** crime scene, DMAC, fluorescence, phytochemicals, tile surface, urine

## Abstract

Forensic science depends on identifying biological fluid stains to maintain DNA for profiling and associating suspects with crime scenes. These stains can include urine and other secretions; however, presumptive and confirmatory tests often target specific fluids and might damage DNA because of destructive chemical processes. Traditional reagents like 4‐dimethylaminocinnamaldehyde (DMAC) allow for quick screening but have low specificity and sensitivity in complex crime scene matrices. Hence, there is a necessity for a universal, highly sensitive, and non‐destructive technique for the accurate analysis and differentiation of bodily fluids. In this study, we present an eco‐friendly technique for synthesizing silver nanoparticles (AgNPs) and gold nanoparticles (AuNPs) utilizing extracts from the stems of *Sclerocarya birrea* and *Moringa oleifera* as reducing and stabilizing agents in aqueous medium. These nanoparticles were employed as alternative materials for urine analysis at the crime scene. Various characterization techniques, including Fourier‐transform infrared spectroscopy, X‐ray diffraction (XRD), transmission electron microscopy (TEM), and spectrophotometry, were used to analyse the nanoparticles. XRD results indicated a face‐centred cubic crystalline structure, while microscopy observations showed that the nanoparticles were mainly spherical, with some in hexagonal and polyhedral shapes. Fluorescence spectroscopy revealed that AgNPs and AuNPs significantly improved the excitation and emission signals of DMAC in urine analysis when compared to plant extracts alone. Furthermore, electrophoresis gel and nanophotometer analysis showed enhanced DNA recovery and less degradation after nanoparticle‐assisted urine testing. These results indicate that these nanoparticles function as non‐toxic signal enhancers and protective agents, thereby enhancing sensitivity, specificity, and DNA preservation in forensic analysis.

## Graphical abstract

This study highlights how gold and silver nanoparticles (AuNPs and AgNPs) were prepared from *M* *oringa* *oleifera* and *Sclerocarya birrea* plant extracts and used for the analysis of urine components in the detection of urine at crime scenes. Both types of nanoparticles were characterized using techniques such as tranmission electron microscopy and UV–Visible spectroscopy whilst maintaining DNA integrity.

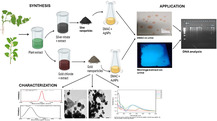

## 1 Introduction

The identification of biological fluids in forensic investigations is a crucial step in crime scene analysis, as it facilitates the recovery and preservation of DNA evidence for purposes of human identification and event reconstruction [1]. The biological fluids typically found at crime scenes include blood, semen, saliva, vaginal secretions, sweat, and urine, all of which provide DNA for the identification of people as well as contextual information that assists in reconstructing the timeline and details of the criminal act [2]. Among these, urine is frequently neglected, even though it holds significant forensic importance in cases like sexual assault, harassment, and contamination incidents connected to assaults, where small traces of urine can yield essential DNA and toxicological evidence [3]. Nevertheless, accurately analyzing and detecting urine is difficult due to its diluted biochemical composition and the shortcomings of existing presumptive tests. Current forensic methods depend on biochemical and colorimetric reagents that specifically target various components in urine. For instance, 4‐dimethylaminocinnamaldehyde (DMAC) is commonly utilized because it quickly reacts with urea in alkaline environments, resulting in a fluorescent product [4, 5]. Furthermore, traditional tests like the Jaffe reaction focus on detecting creatinine compound in urine. Despite this, research indicates that methods for detecting creatinine are hindered by matrix interference and limited selectivity, whereas DMAC‐based assays may cross‐react with other biological fluids that contain amines and compounds similar to urea, leading to false positives and DNA degradation due to their destructive properties [6, 7].

Previous and recent studies have shown that nanoparticle‐based sensing systems can detect urine biomarkers like creatinine, especially employing gold and silver nanoparticles, with detection relying on plasmonic aggregation and color changes [8]. However, several of these systems necessitate intricate functionalization, sample pretreatment, and costly reagents, which restrict their applicability in forensic body fluid investigations. Urine was chosen as the focus of this study because of its notable forensic significance and its relatively limited use compared to other biological fluids. Urea, a major component in urine, is a chemically stable and abundant analyte, providing benefits over other metabolites like creatinine and uric acid found in urine [9, 10, 11]. The constant presence and high levels of urea in urine make it an ideal molecular focus for analytical detection, especially in reactions with 4‐dimethylaminocinnamaldehyde (DMAC), which has been extensively used in forensic and biochemical tests involving urea analysis. Ismael et al. (2022) developed a highly sensitive fluorometric technique utilizing silver nanoparticles to identify and measure urea in actual urine samples through interactions between nanoparticles and urea [12]. Alula et al. (2018) developed a colorimetric sensor using citrate‐capped silver nanoparticles to achieve quick, specific, and highly sensitive detection of creatinine in urine through analyte‐induced aggregation of the nanoparticles. This system allowed for quantification in just a few minutes with exceptional sensitivity (nanomolar detection limit) by measuring optical variations in nanoparticle plasmon resonance [8]. Elmasry et al. (2023) developed a dual‐signal sensing system that integrates fluorescent carbon dots with plasmonic silver nanoparticles generated in situ for the sensitive detection of urea through pH changes triggered by urease [13].

This research introduces a green nanotechnology‐based technique to improve the detection and analysis of urine samples and develops a novel biosensor utilizing plant extracts as an environmentally friendly and cost‐effective alternative for urine analysis to address ongoing issues associated with conventional techniques of urine analysis that compromise DNA integrity and require costly, laborious procedures for analyzing urine samples. Silver nanoparticles (AgNPs) and gold nanoparticles (AuNPs) were synthesized using extracts from the stems of *Sclerocarya birrea* and *Moringa oleifera*, and these were employed as enhancers to enhance the performance of DMAC by enhancing its sensitivity, specificity and minimizing its DNA‐damaging toxicity. In this research, silver and gold nanoparticles derived from *Sclerocarya birrea* and *Moringa oleifera* stem extracts were used to enhance the sensitivity and specificity of DMAC for better analysis of urine. *Moringa oleifera* and *Sclerocarya birrea* water extracts were further tested for urine analysis as a potential cost‐effective and nondestructive alternative technique for urine detection at a crime scene. The synthesis of silver and gold nanoparticles was accomplished by the utilization of a green chemistry method, wherein water was used as the solvent for extracting compounds from *Moringa* and *Marula* stem extracts. These plants are rich in phytochemicals such as polyphenols, alkaloids, and flavonoids and are known for their ability to act as reducing and capping agents, facilitating the reduction of Au (III) to Au (0) and Ag(I) to Ag (0). The decision to opt for the green chemistry method was based on its eco‐friendliness and cost‐effectiveness, as opposed to chemical and physical procedures that include hazardous substances and demand substantial energy consumption [14, 15, 16, 17, 18, 19, 20].

**FIGURE 1:**
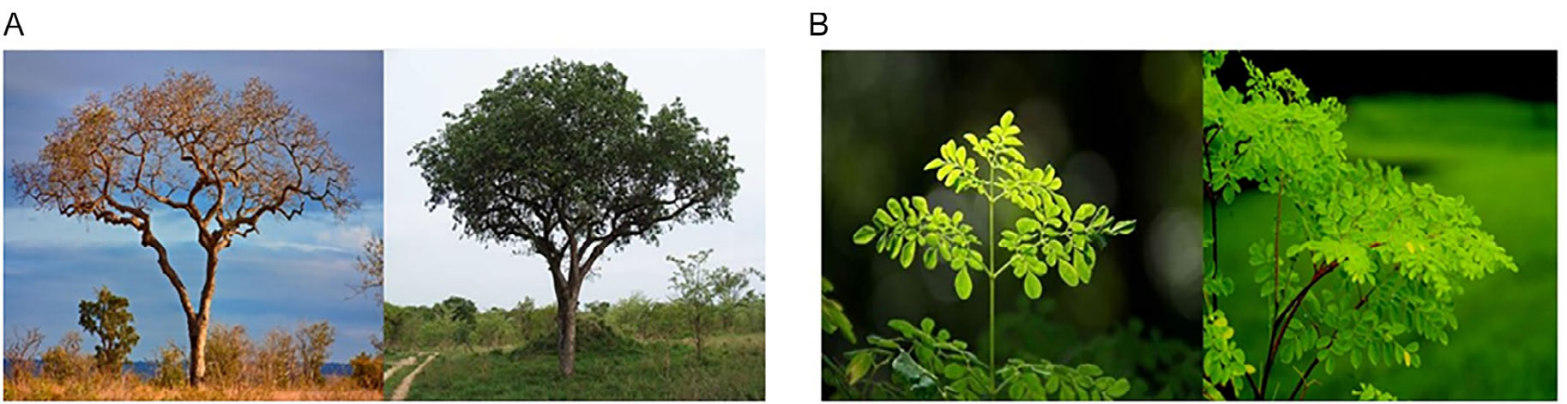
(A) *Sclerocarya birrea* tree and (B) *Moringa Oleifera* plant.

Comparative studies were conducted to assess whether the plant‐based biosensor could serve as an affordable and alternative material for DMAC in urine analysis. In contrast to conventional nanoparticle‐based detection techniques, which rely on complex preparation steps and less effective biomarkers, this approach offers a simpler, label‐free, eco‐friendly, and cost‐effective technique that is safer for long‐term use. The synthesized AuNPs and AgNPs were analyzed for their morphological and optical properties using various techniques, including transmission electron microscopy (TEM), Fourier‐transform infrared spectroscopy, X‐ray diffraction (XRD), Zetasizer, spectrophotometer, and ultraviolet–visible spectroscopy. The results from XRD validated the formation of crystalline structures, while TEM analysis showed that the nanoparticles mainly exhibited spherical, triangular, and rod‐like geometries. These results highlight the potential of plant extract biosensor, plant‐based AgNPs, and AuNPs as effective signal enhancers, providing a nondestructive and environmentally friendly method for enhanced urine analysis in forensic applications. The *Sclerocarya birrea* tree and *Moringa oleifera* plant used for the synthesis of AgNPs and AuNPs are depicted below in Figure 1 [21].

## 2 Methods and Materials

### 2.1 Materials

Silver nitrate (AgNO_3_, 99%), tryptamine, DMAC, and chloroauric acid trihydrate (HAuCl_4_·3H_2_O, 98%) were purchased from Sigma Aldrich, SA. Urine samples were collected from voluntary donors in Johannesburg. *Marula* stems were harvested from Limpopo Province in South Africa. Stems of *Moringa* were obtained from a nursery in Johannesburg, Gauteng Province, South Africa. Deionized water was used throughout the studies, and all chemicals and reagents utilized were of analytical grade. No further purification was performed on the chemicals before their use.

### 2.2 Preparation of *Sclerocarya birrea* Extract, *Moringa oleifera* Stem Extract, and Silver Nanoparticles Derived from both Plants

The stem samples were first cleaned with tap water and then rinsed with distilled water to eliminate surface contaminants and impurities. The cleaned stems were subsequently chopped into smaller parts and left to air‐dry for 3–4 weeks. Once fully dried, they were ground into a fine powder with an electric grinder and sifted through a 20‐mesh screen to achieve a consistent particle size. Active polar compounds were extracted into the aqueous phase by the maceration technique. A precise mass balance was used to weigh 20 g of freshly dried and pulverized *Marula* and *Moringa* stem powder separately. Approximately 20 g of plant powders were added to separate 500 mL of distilled water and stirred on a hotplate (LABOTEC MR Hei‐Tec) at 25°C for 2 h to thoroughly extract active polar compounds from *Sclerocarya birrea* and *Moringa oleifera.* After cooling to room temperature, they were filtered through Whatman Grade 3 filter paper (HW 90 mm). The resultant extracts were refrigerated at 4°C until further use. Approximately 20 mL of the stem extracts were combined with 0.5 M silver nitrate solution in different beakers and heated on a hot plate at a temperature of 60°C–70°C for 20 min. The effective synthesis of nanoparticles was confirmed by a color change from red to black. To reduce the agglomeration of nanoparticles, the mixtures were cooled in an incubator shaker for 2 h at a temperature of 25°C and a speed of 120 rpm. Subsequently, the solutions were centrifuged twice at 500 × 10 rpm for 10 min. The supernatants were discarded, and the pellets were rinsed three times with ethanol and distilled water, 50:50 separately. Subsequently, the purified pellets were dried in an oven at 70°C for 2 h [22, 23, 24].

### 2.3 Preparation of Gold Nanoparticles Derived from *Sclerocarya Birrea* Stem Extract and *Moringa oleifera* Stem Extract

Gold nanoparticles (AuNPs) were synthesized by the procedures reported by Boruah et al. and Alhumaydhi et al. [25, 26], with further modifications. Approximately 0.330 M gold chloride solution was prepared by dissolving 0.11 g of gold salt in distilled water in a 1 L volumetric flask. Subsequently, equal amounts (50 mL each) of stem extracts and a freshly prepared 0.330 M aqueous solution of HAuCl_4_·3H_2_O were mixed in separate beakers, resulting in a total volume of 100 mL each of stem extracts and gold chloride solution. The resultant mixture was continuously stirred on a magnetic stirrer for 6 h at 25°C to achieve a homogenous solution. The alteration in color signifies the bioreduction of Au^3+^ ions to Au^0^. Following the heating, the resultant solutions were cooled and stirred in an incubator shaker for 2 h to reduce agglomeration of gold nanoparticles. Gold nanoparticles were subsequently acquired by centrifugation, conducted twice at 500 × 10 rpm for 10 min. The supernatants were removed separately, and the resultant pellets were washed three times with distilled water and ethanol 50:50. The purified pellets were further dried in an oven at 70°C for 60 min [27].

### 2.4 Urine Analysis Method and Preparation of 4‐Dimethylaminocinnamaldehyde (DMAC)

Approximately 5 μL of human urine samples were deposited onto both filter paper and tile surfaces, then left to air dry. The resultant stains were analyzed using a few drops of DMAC reagent (at concentrations of 0.1% and 0.05%), along with *Marula* extract, *Moringa* extract, and a nanoparticles‐based DMAC reagent in a laboratory setting under UV light at wavelengths of 250 and 350 nm to evaluate the differences. The reactions were observed at various time intervals. The DMAC solution was prepared according to the procedure outlined by Sandy et al. (2012) [10]. The reagent was dissolved in ethanol at concentrations of 0.05 and 0.1 g for every 100 mL to create 0.05% and 0.1% (w/v) solutions, respectively. Following this, 3 mL of concentrated hydrochloric acid (37% HCl) was combined with 27 mL of each solution to reach a total volume of 30 mL.

### 2.5 Characterization

Gold nanoparticles (AuNPs) and silver nanoparticles (AgNPs) synthesized from the stem extracts of *Sclerocarya birrea* and *Moringa oleifera* were thoroughly characterized using various analytical techniques. To identify the functional groups in the plant extracts that serve as reducing and capping agents, FTIR spectroscopy (PerkinElmer Spectrum 100) was utilized, with KBr pellet preparation allowing for precise mid‐infrared measurements (4000–400 cm^−1^). The structural and crystallographic characteristics were studied using XRD (PANalytical EMPYREAN) with Cu Kα radiation (*λ* = 1.54060 Å) over a broad scan range at a controlled temperature. Optical absorption profiles of nanoparticles were measured using UV–Vis spectroscopy (PerkinElmer Lambda 25/Shimadzu UV2450) within the range of 200–900 nm. Particle size distribution and surface charge were analyzed using a Zetasizer Nano ZS through dynamic light scattering (DLS) and measurements of electrophoretic mobility. Morphological and elemental composition of AgNPs and AuNPs were conducted with TEM (JEOL‐2100) coupled with EDS mapping. High‐resolution scanning electron microscopy (HRSEM) (Zeiss Sigma FE‐SEM) for high‐resolution imaging and surface analysis was used to analyze the surface morphology of AgNPs and AuNPs. The fluorescence characteristics of AgNPs and AuNPs were assessed using a Shimadzu RF‐6000 spectrofluorometer with excitation at 400 nm and emission at 900 nm, which confirmed the optical properties of the nanoparticles.

## 3 Results and Discussion

### 3.1 FTIR Analysis

**FIGURE 2:**
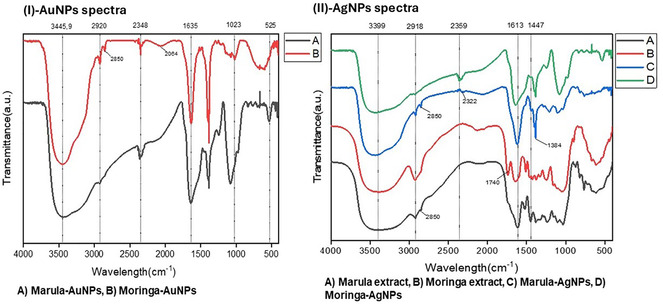
FTIR spectra of gold nanoparticles (I) and silver nanoparticles (II) derived from the stem extracts of *Sclerocarya birrea* (Marula) and *Moringa oleifera*.

Figure 2 displays the FTIR spectra of *Sclerocarya birrea* and *Moringa oleifera* stem extracts, as well as the AgNPs and AuNPs derived from these extracts. The spectra demonstrate that comparable or identical functional groups in both stem extracts facilitated the binding and reduction of Ag^+^ and Au^3+^ ions to AgNPs and AuNPs, respectively. Absorbance peaks were detected within the ranges of 3000–3500, 2800–3000, 1626, 1400–1550, 1380–1403, and 1000–1100 cm^−1^, correlating with the functional groups involved in the reduction of metal salts to nanoparticles. The identified peaks are ascribed to the stretching vibrations of hydroxyl groups in alcohols or phenolic compounds, CH_2_ and CH_3_ groups, aromatic compound C=C bonds, carboxylic acid C=O groups, amide I (CONH_k_) and amide II (CONH) groups, geminal methyl, ether linkages, and C—O or C—O—C functional groups [23]. In the spectra of the comparable silver nanoparticles (AgNPs), these peaks seemed to be absent or had markedly diminished intensity. This suggests that functional groups in these areas are essential for the reduction and stability of AgNPs and AuNPs by subsequent oxidation. In the spectra of gold nanoparticles, these peaks were absent, and some were present and more pronounced than on the AgNPs spectra of both *Sclerocarya birrea* and *Moringa oleifera*. Furthermore, ether linkages, along with C—O and C—O—C functional groups, are linked to flavones, terpenoids, and polysaccharides present in plant biomass. The surface of AgNPs and AuNPs synthesized from plant extracts of medicinal plants has also been found to contain these functional groups [22, 25].

### 3.2 XRD Analysis

**FIGURE 3:**
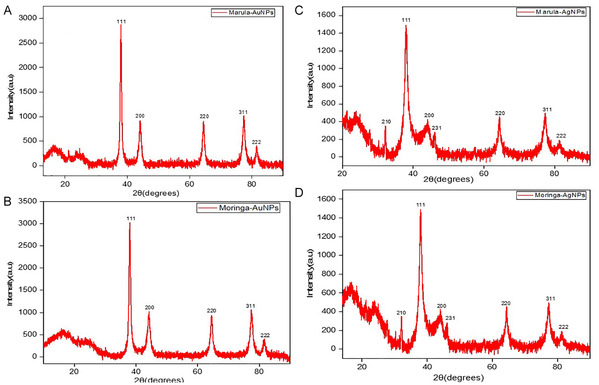
XRD spectra of AgNPs (C and D) and AuNPs (A and B) derived from *Marula* and *Moringa* *oleifera* stem extracts.

The analysis of XRD was used to ascertain the purity and crystalline structure of AgNPs and AuNPs. The spectra validated the purity of the synthesized gold and silver nanoparticles, as illustrated in Figure 3 (AuNPs and AgNPs, respectively). Diffraction peaks observed at 2θ values of 38.07°, 44.29°, 64.45°, 77.43°, and 81.66° correspond to the crystal planes (111), (200), (220), (311), and (222) of metallic gold, as depicted in Figure 3A,B. The observed peaks confirm the formation of a face‐centered cubic (FCC) structure in the AuNPs, a process enabled by the reduction of Au^+^ and Ag^+^ ions by *Moringa* and *Marula* extracts. The observed peaks exhibit a strong correlation with the reference powder data from JCPDS file number 04–0783. Furthermore, the *Moringa* and *Marula* extracts within AuNPs demonstrated an amorphous structure, as indicated by a low‐intensity peak at 2θ = 24.13° (002) in Figure 3A,B. The indeterminate characteristics can be ascribed to the variety of chemical compounds found within the extracts of *Moringa oleifera* stems [25, 28]. The XRD pattern of the synthesized AgNPs, *Marula*‐AgNPs, and *Moringa*‐AgNPs in Figure 3C,D exhibited Bragg reflection peaks at 2θ values of 32.16°, 38.12°, 44.3°, 46.21°, 64.42°, 77.45°, and 84°. The observed peaks align with the (210), (111), (200), (231), (220), (311), and (222) planes of pure silver, which are indexed to a FCC crystalline structure of metallic silver, as corroborated by JCPDS file No. 04–0783. The indeterminate characteristics of the *Moringa oleifera* stem and *Marula* stem extracts within the AgNPs were clearly illustrated by a broad, low‐intensity peak at 2θ = 23.98° (002) in Figure 3C,D. The XRD spectra demonstrated the successful synthesis of gold and silver nanoparticles (AuNPs and AgNPs) derived from the stem extracts of *Marula* and *Moringa oleifera*, both demonstrating crystalline structures. Several low‐intensity unassigned peaks, illustrated in Figure 3A–D, are ascribed to the crystallization of bio‐organic molecules on the surfaces of the AuNPs and AgNPs from the stem extract [29, 30]. The crystalline dimensions of the AgNPs and AuNPs were determined through an analysis of the broadening of the significant peaks at 2θ = 38.07° (111) for *Moringa*‐AgNPs and 2θ = 37.73° (111) for *Moringa*‐AuNPs, and 2θ = 38.07° (111) for *Marula*‐AgNPs and 2θ = 37.73° (111) for *Marula*‐AuNPs, utilizing the Debye–Scherrer equation

(1) d=kλ/(β×cosθ)

*d* represents the average crystalline size, *k* denotes the Scherrer constant (0.9), *λ* signifies the X‐ray wavelength (0.154 nm), and *β* indicates the full width at half maximum of the peaks measured in radians. The determined average crystalline sizes for AgNPs and AuNPs from *Marula* and *Moringa* were found to be 11.12 and 18.28 nm, respectively. Both plants have similar values of crystalline sizes, as they have similar plane fractions [31, 32].

### 3.3 UV Spectroscopy Analysis (UV–Vis) Analysis

**FIGURE 4:**
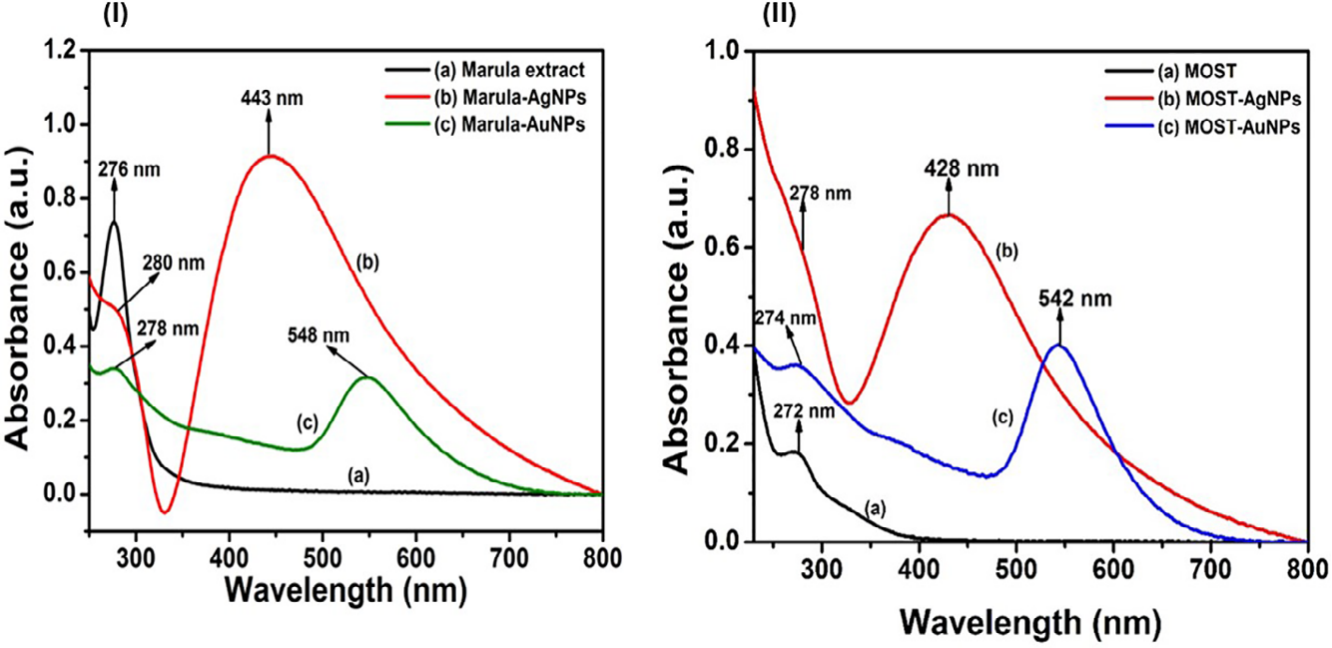
UV–visible spectra of silver nanoparticles derived from *Marula* extract (Ib and IIb) and (Ic and IIc) gold nanoparticles derived from *Moringa oleifera* stem extract (MOST).

UV–VIs spectroscopy is a vital technique for assessing the formation and stability of metal nanoparticles in aqueous solutions. The known correlation between the absorbance properties of UV–visible light, size, and configuration of the absorbing material facilitates the assessment of nanoparticle morphology in suspension [33, 34]. The Ag and Au nanoparticles synthesized from *Moringa oleifer*a water stem extract and *Marula* extract were analyzed using UV–VIs absorption spectroscopy. Figure 4 shows the results of UV–Vis from *Moringa oleifera* and *Marula* stem extracts. The UV–Vis absorption spectra of AgNPs (Figure 4Ib,IIb) derived from both plants display two broad peaks between 428 and 443 nm and a low‐intensity peak at 278 and 280 nm, thereby confirming the synthesis of AgNPs, with plant extracts serving as both stabilizing and reducing agents. During the synthesis, the plant extract's phytochemicals, such as polyphenols, flavonoids, and alkaloids, act as reducing agents, converting Au^3+^ ions to Au^0^ and Ag^+^ to Ag^0^. The synthesis of gold nanoparticles (AuNPs) was confirmed by a notable color change from yellow to ruby red, a defining attribute of ultrasmall colloidal AuNPs, while silver nanoparticles were confirmed by a notable color change to black. The predominant formation of spherical silver nanoparticles or an increasing proportion of anisotropic particles over time was indicated by the observation of a maximal absorbance peak at 428 and 443 nm. The synthesis of AuNPs from *Marula* and *Moringa* (Figure 4Ic,IIc) was validated by two absorption peaks at 542 and 548 nm, indicative of the absorption characteristics of metallic gold nanoparticles [35, 36]. The peaks identified within the 200–330 nm range (Figure 4Ia,IIa) correspond to phytochemicals extracted from *Moringa oleifera* and *Marula*. The anticipated outcome is due to the lack of nanoparticles in the aqueous extracts, which negates any absorbance associated with nanoparticles. Silver and gold nanoparticles are recognized for their distinctive reddish‐brown color in aqueous solution, attributed to the activation of surface plasmon vibrations. The emergence of this coloration in the reaction vessels verifies the synthesis of silver and gold nanoparticles. Silver nitrate and gold chloride function as salt precursors for the synthesis of silver and gold nanoparticles, utilizing distinct qualities such as strong conductivity, catalytic activity, and chemical stability [37, 38, 39, 40].

### 3.4 Particle Size Distribution and Zeta Potential Analysis

#### 3.4.1 Particle Size Distribution Analysis

**FIGURE 5:**
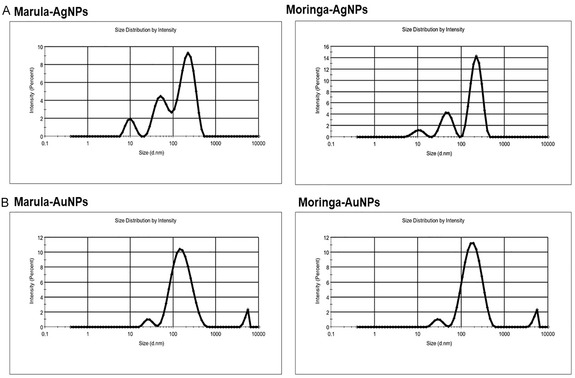
Particle size distribution of as‐synthesized AgNPs (A) and AuNPs (B) derived from *Marula* and *Moringa oleifera* stems water extracts.

The measurements of particle size acquired through DLS denote the hydrodynamic diameter (nm) of nanoparticles within a liquid dispersion. The synthesized silver nanoparticles (AgNPs) derived from *Sclerocarya birrea* and *Moringa oleifera* stem extracts (Figure 5) demonstrated hydrodynamic sizes of between 10 and 80 nm, whereas the gold nanoparticles (AuNPs) were measured at 15 and 95 nm, respectively. The DLS results obtained were comparable to the previously reported sizes of 77 and 105 nm for different green synthesized nanoparticles, as reported by Nune et al. and Lediga et al. 0.329 [41, 42]. In this study, the synthesized silver nanoparticles (AgNPs) demonstrated polydispersity index (PDI) values of 0.329 for *Moringa* AgNPs and 0.363 for *Sclerocarya birrea* (*Marula*) AgNPs. Similarly, the gold nanoparticles (AuNPs) exhibited PDI values of 0.341 for *Marula* and 0.325 for *Moringa*, respectively. The values presented suggest a significant level of polydispersity, accompanied by a negligible likelihood of aggregation. A PDI value surpassing 0.5 indicates the occurrence of nanoparticle aggregation within the solution, resulting in instability. Consequently, these findings confirm the effective stabilization of the AgNPs synthesized from the selected medicinal plant species [43].

#### 3.4.2 Zeta Potential Analysis

**FIGURE 6:**
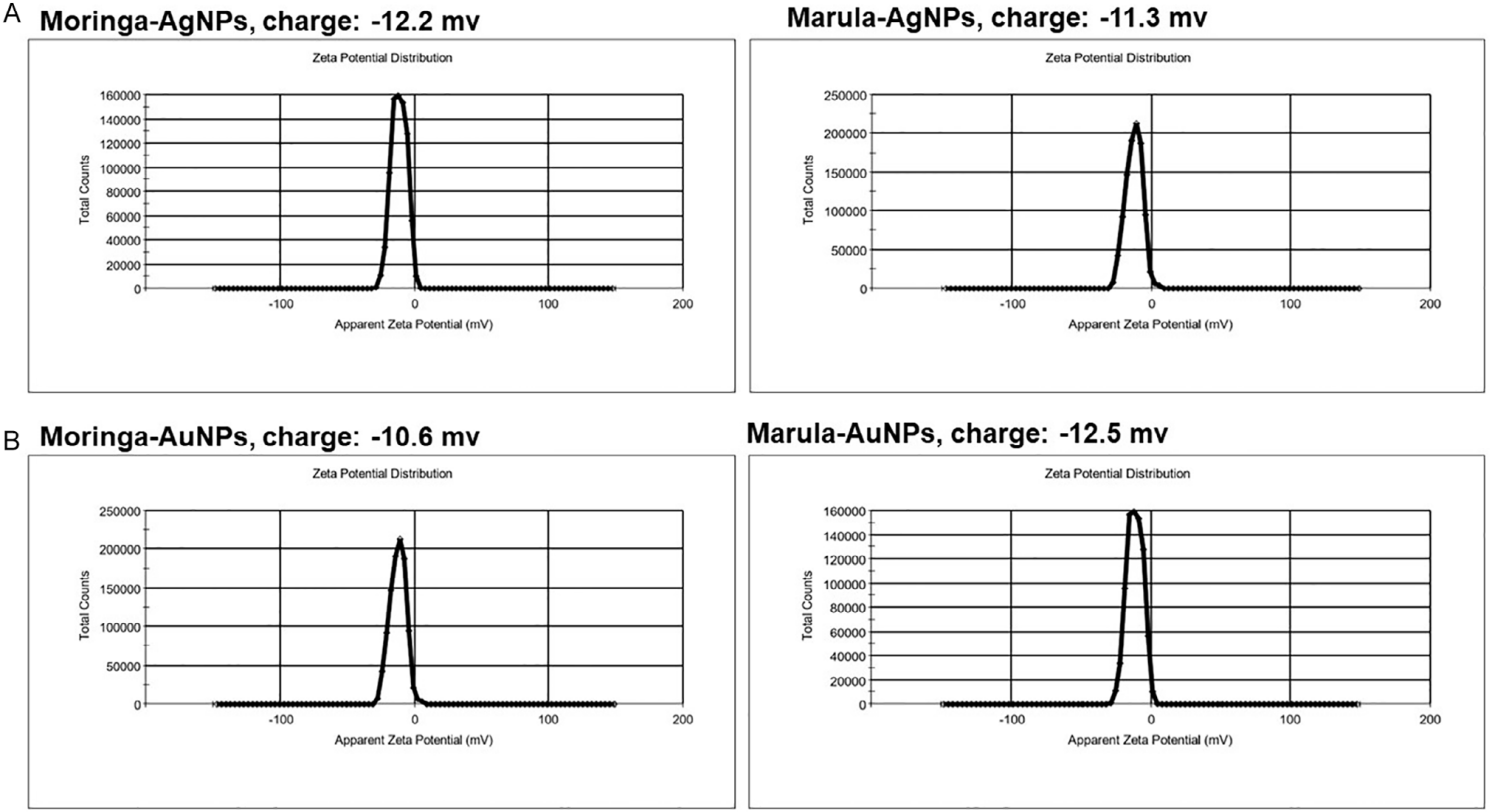
Zeta potential of as‐synthesized AgNPs (A) and AuNPs (B) derived from *Marula* and *Moringa oleifera* stems' water extracts.

The surface zeta potential is a crucial parameter to measure the stability of nanoparticle dispersions in aqueous environments and acts as an indicator of their long‐term stability [44]. Nanoparticles characterized by a high zeta potential demonstrate significant electrostatic repulsion, thereby minimizing the potential for aggregation. The silver nanoparticles (AgNPs) derived from *Sclerocarya birrea* (*Marula*) and *Moringa* extracts exhibited negative charges, displaying zeta potentials of −11.3 and −12.2 mV, respectively, as shown in Figure 6. Comparably, the gold nanoparticles (AuNPs) derived from *Marula* and *Moringa* exhibited zeta potentials of −12.5 and −10.6 mV, respectively. The values demonstrate efficient electrostatic repulsion, thereby reducing the likelihood of nanoparticle aggregation [45].

### 3.5 TEM Analysis

**FIGURE 7:**
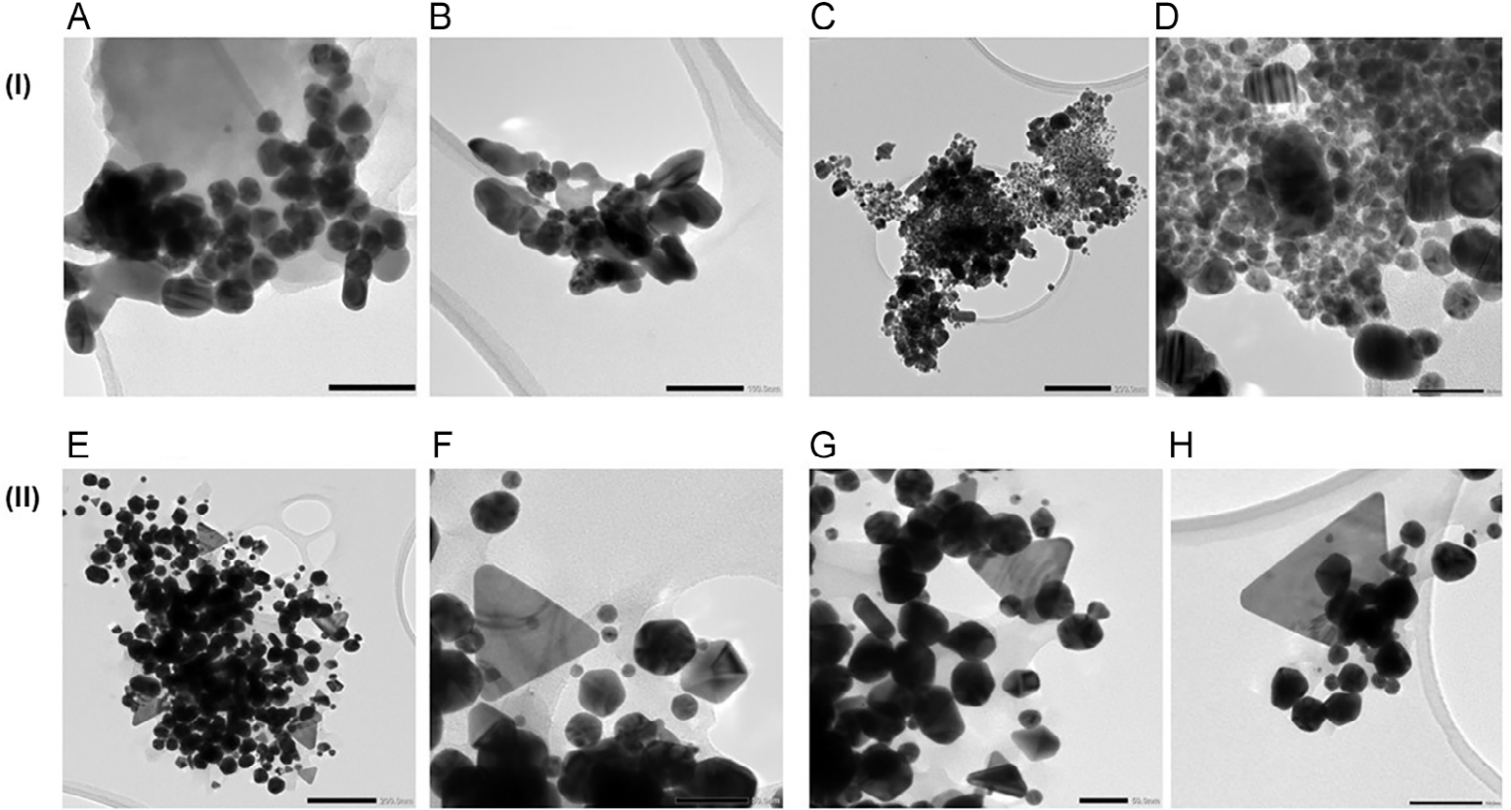
TEM micrographs for AgNPs (I) and AuNPs (II) derived from *Sclerocarya birrea* and *Moringa oleifera* stem extracts.

Figure 7 illustrates the TEM micrographs of the synthesized gold (AuNPs) and silver (AgNPs) nanoparticles synthesized from *Sclerocarya birrea* and *Moringa oleifera*, displayed at various magnifications. The micrographs of plant‐derived AgNPs and AuNPs presented in Figure 7IA–D are for *Marula* and *Moringa oleifera* AgNPs, and Figure 7IIE–H are for *Marula* and *Moringa oleifera* AuNPs, respectively. The micrographs illustrate a variety of morphologies, encompassing spherical, rod‐shaped, cubic, cylindrical, irregular, and other anisotropic forms, with an average particle size spanning from 10 to 50 nm. Similarly, the TEM micrographs of plant‐derived AuNPs from *Marula* and *Moringa*, as illustrated in Figure 7IIE–H, demonstrate their crystalline nature, depicting a range of shapes including spherical, nanorods, cubic, triangular, decahedral, icosahedral, and hexagonal plate structures, with dimensions ranging from 5 to 30 nm. These results correlate with the average nanoparticle size discovered by Zeta Sizer measurements, about 100 nm. TEM provides the size of individual nanoparticles, while Zeta Sizer determines the average hydrodynamic diameter, including aggregated particles. Thus, compared to those observed under TEM, nanoparticles measured with the Zeta Sizer appear larger, but they fall in the 100 nm range. The morphological characteristics observed are indicative of nanoparticles synthesized from plant extracts [46, 47]. The nanoparticles demonstrate a well‐dispersed nature, showing minimal signs of agglomeration, which is further corroborated by the zeta potential analysis.

### 3.6 Spectrophotometer Analysis

**FIGURE 8:**
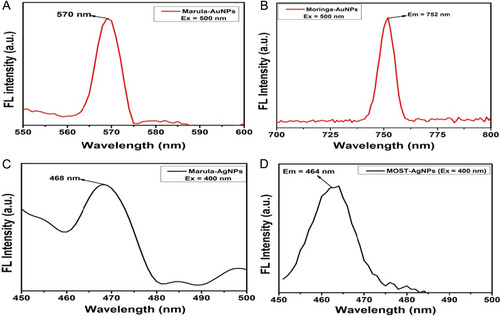
Fluorescence spectra of AuNPs (A and B) and AgNPs (C and D), derived from *Marula* and *Moringa oleifera* stem extracts.

The emission fluorescence properties for silver (AgNPs) and gold (AuNPs) nanoparticles derived from *Moringa oleifera* and *Marula* stem extracts were analyzed, with the results illustrated in Figure 8. The emission and excitation characteristics of gold and silver nanoparticles synthesized from plant extracts are influenced by their size and morphology [48]. The Zetasizer analysis indicates that the nanoparticles have hydrodynamic diameters between 10 and 90 nm. As the size of the nanoparticles increases, the SPR peak shifts to longer wavelengths (redshift) as a result of enhanced plasmonic coupling and radiative damping. The emission and excitation intensity are predominantly influenced by surface plasmon resonance (SPR), which is significantly contingent upon the size and shape of nanoparticles, as evidenced by UV–VIs spectroscopy [49, 50]. However, the emission spectra for AgNPs and AuNPs exhibit notable differences due to the difference in optical properties of silver and gold. The silver nanoparticles derived from *Moringa* and *Marula* demonstrated a higher excitation peak under UV light, which is referred to as the SPR peak, in comparison to their gold nanoparticle peaks. SPR occurs when conduction electrons on the nanoparticle surface oscillate in resonance with incident light, leading to amplified electromagnetic fields at the nanoparticle interface. This effect enhances both dispersion and light absorption [51]. The emission spectra linked to SPR result from electron mobility in nanoparticles and the recombination of electrons between the sp band and d band holes. The emission peaks for silver nanoparticles from *Marula* and *Moringa oleifera* are less intense and broader compared to the gold nanoparticles spectra, which is because silver nanoparticles exhibit a greater tendency to quench fluorescence, attributed to their elevated plasmonic energy. Silver nanoparticles (AgNPs) exhibited a more pronounced SPR compared to gold nanoparticles when exposed to UV–VIs light, attributed to their elevated free electron density. This explains the higher absorbance peak intensity of AgNPs in the UV–Vis region 400–450 nm, as indicated in Figure 4Ib,IIb [52].

Fluorescence quenching of AgNPs is due to their metallic surface interacting with fluorescent molecules such as phytochemicals from *Marula* and *Moringa* via a mechanism known as nonradiative energy transfer (FRET) [52, 53]. This results in decrease in fluorescence emission intensity, as shown in Figure 8. Gold nanoparticles demonstrated reduced SPR absorbance in the UV–Vis range of 520–550 nm, as shown in Figure 4Ic,IIc (Ic and IIc); however, they facilitate enhanced radiative energy transfer to fluorescent molecules and result in higher emission fluorescence, as shown in Figure 8. Extracts from plants such as *Marula* and *Moringa* are rich in phenolic compounds, flavonoids, and proteins, serving as natural reducing and stabilizing agents. The interaction of these biomolecules with gold nanoparticles may lead to a stronger binding, resulting in the formation of a protective layer that enhances fluorescence emission. Silver nanoparticles exhibit a greater affinity for these biomolecules, leading to the quenching of fluorescence signals [48, 54].

### 3.7 HRSEM Analysis

**FIGURE 9:**
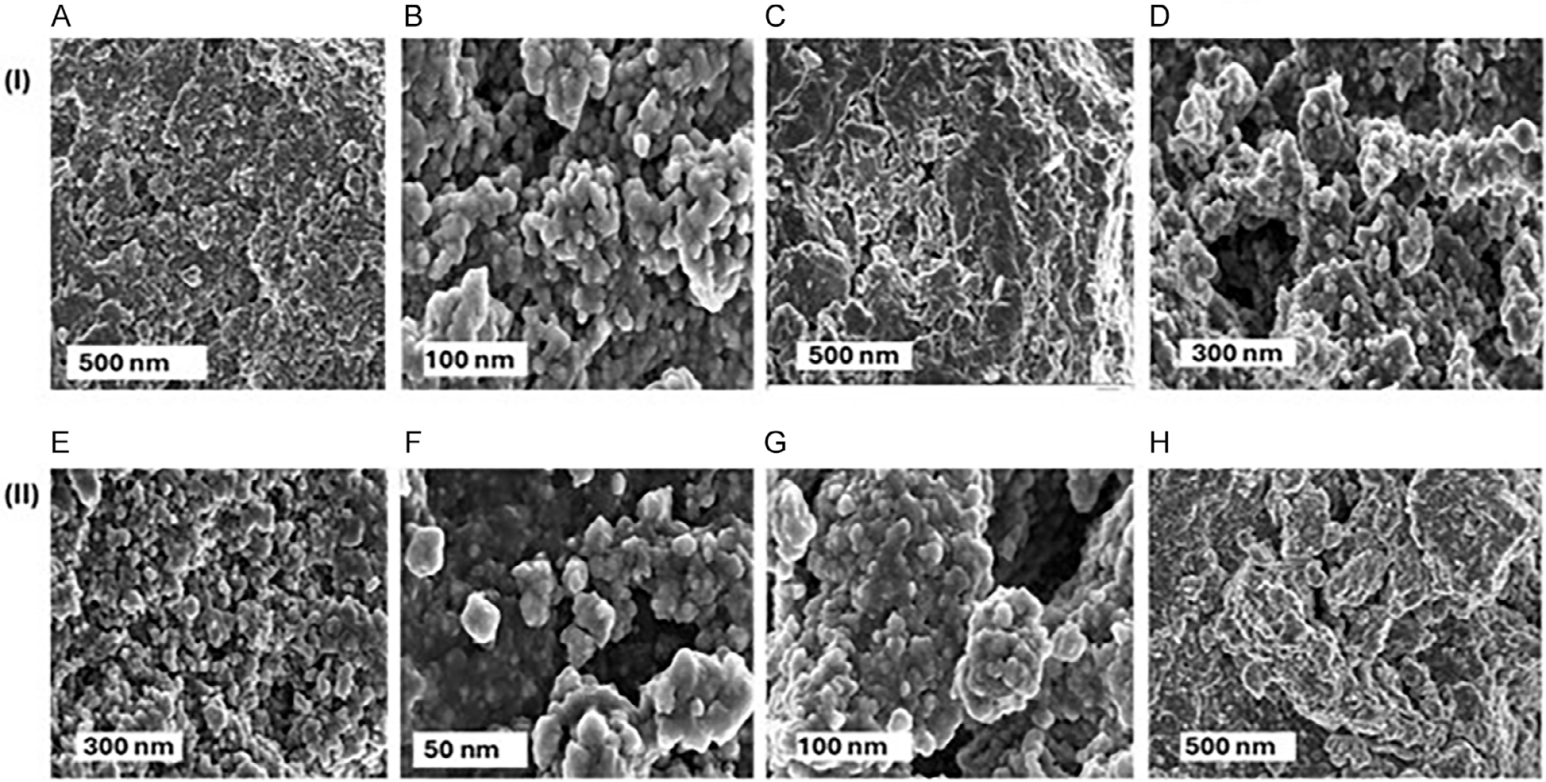
HRSEM micrographs of AgNPs (I) and AuNPs (II) derived from *Sclerocarya* *birrea* and *Moringa Oleifera* stem extracts.

Gold nanoparticles (AuNPs) and silver nanoparticles (AgNPs) synthesized from *Sclerocarya birrea* and *Moringa oleifera* stem extracts were subjected to HRSEM to analyze their surface morphology. The results are shown in Figure 9. Scanning electron microscopy (SEM) analysis demonstrated a homogeneous distribution of silver (AgNPs), Figure 9IA–D, and gold (AuNPs) nanoparticles, Figure 9IIE–H, synthesized from *Marula* and *Moringa* extracts, respectively, indicating efficient stabilization by plant‐derived capping agents [55]. The Ag and Au nanoparticles primarily exhibited a spherical morphology with little agglomeration, and their sizes varied within the 1–90 nm range. The presence of bigger silver and gold nanoparticles from the micrographs of AgNPs and AuNPs derived from *Marula* and *Moringa* may result from the agglomeration of smaller particles, as indicated by SEM observations. Moreover, phytochemicals derived from plant extracts, serving as both reducing and capping agents such as alkaloids, polyphenols, and flavonoids, contribute to particle size by adhering to nanoparticle surfaces, thus affecting their overall size [56].

### 3.8 Analysis and Detection of Urine Utilizing *Marula* Stem Water Extract, *Moringa oleifera* Stem Extract Tryptamine, 4‐Dimethylaminocinnamaldehyde (DMAC), Silver, and Gold Nanoparticles. The Analysis was Conducted under UV Light and Ordinary Light

**FIGURE 10:**
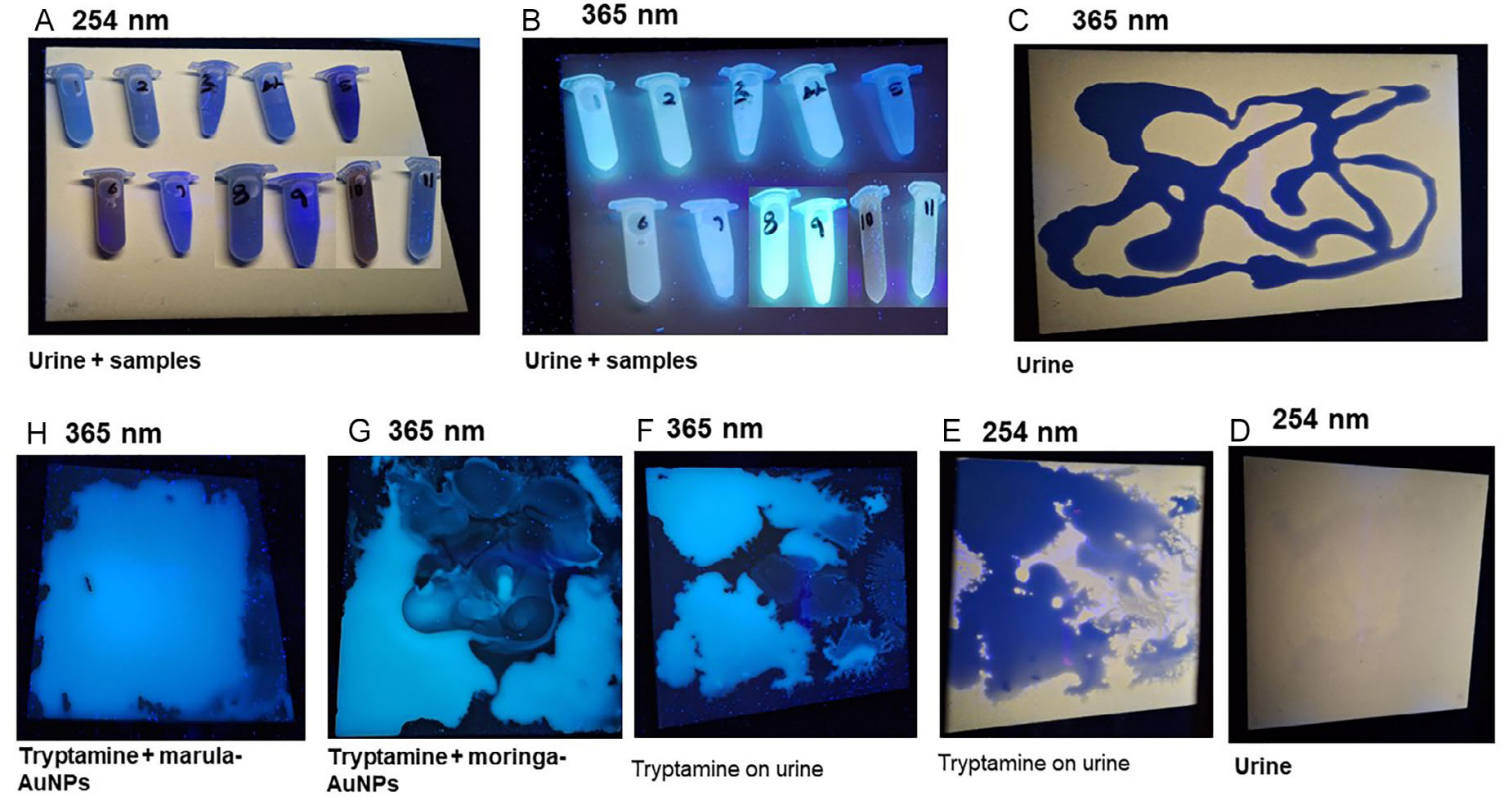
Analysis and detection of human urine samples by plant extracts and tryptamine compound under UV light at 254 and 365 nm.

**FIGURE 11:**
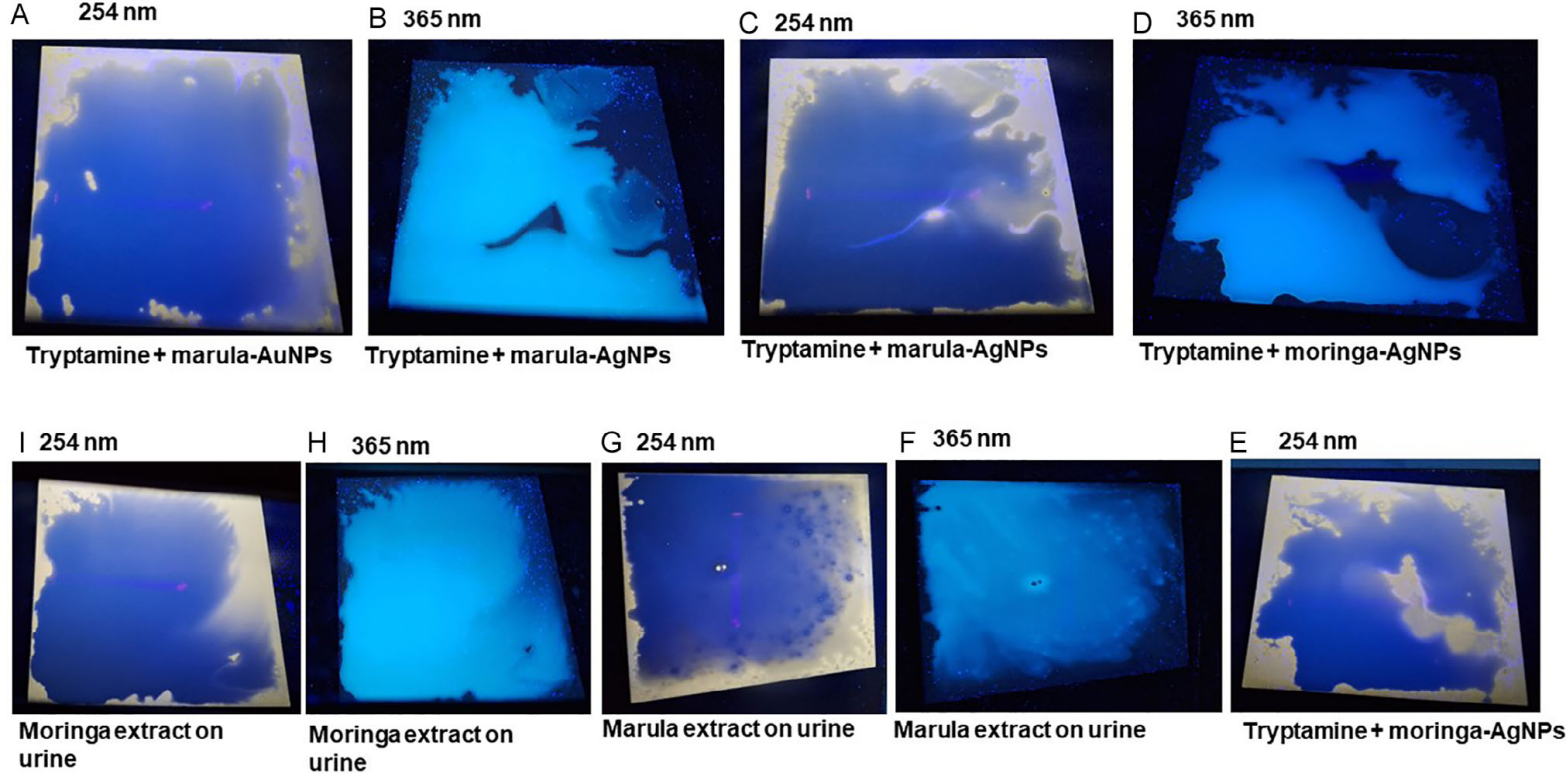
Urine stain analysis on tile surface using tryptamine + nanoparticles, *Marula*, and *Moringa* extract under UV light at 254 and 365 nm.

This study assessed the efficacy of various reagents in improving the identification of urine stains on tile surfaces and paper towels, which are frequently encountered in forensic investigations. Initial analysis indicated that untreated urine stains showed only faint fluorescence under UV light, particularly at 365 nm, as presented in Figure 10D, which is not sufficient for reliable analysis. The incorporation of tryptamine and tryptamine‐coated silver and gold nanoparticles led to a notable increase in fluorescence intensity (Figure 10E–H), highlighting the effectiveness of phytochemical‐assisted nanomaterials in detecting urine. Subsequent analysis (Figure 11) indicated that extracts from *Sclerocarya birrea* and *Moringa oleifera*, along with tryptamine, exhibit inherent fluorescence, with a stronger emission at 365 nm, validating their potential as efficient, nondestructive detection agents under UV light.

**FIGURE 12:**
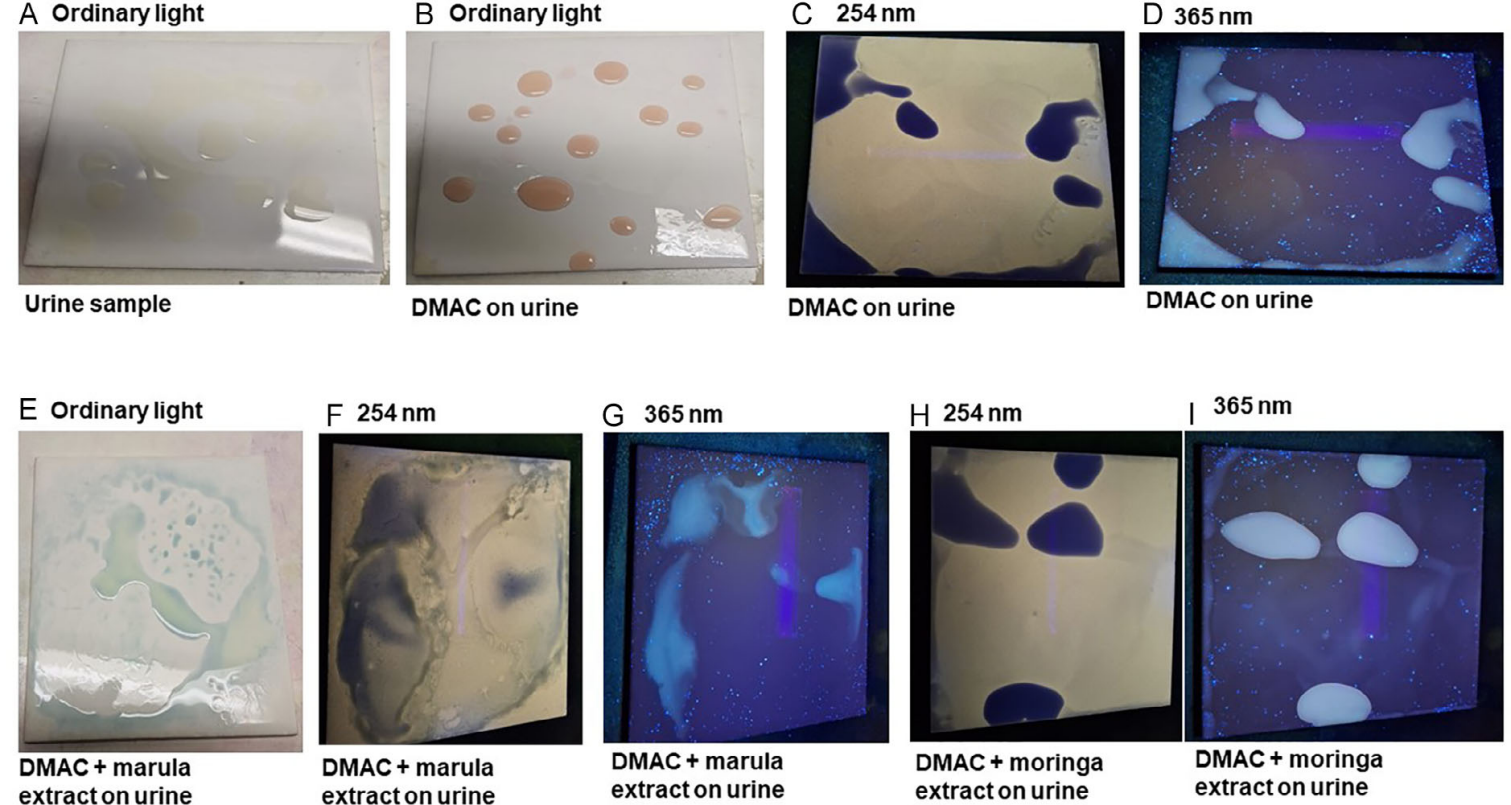
Urine analysis on the tile surfaces using DMAC and DMAC‐based AgNPs and AuNPs was viewed under UV light at 254 and 365 nm.

**FIGURE 13:**
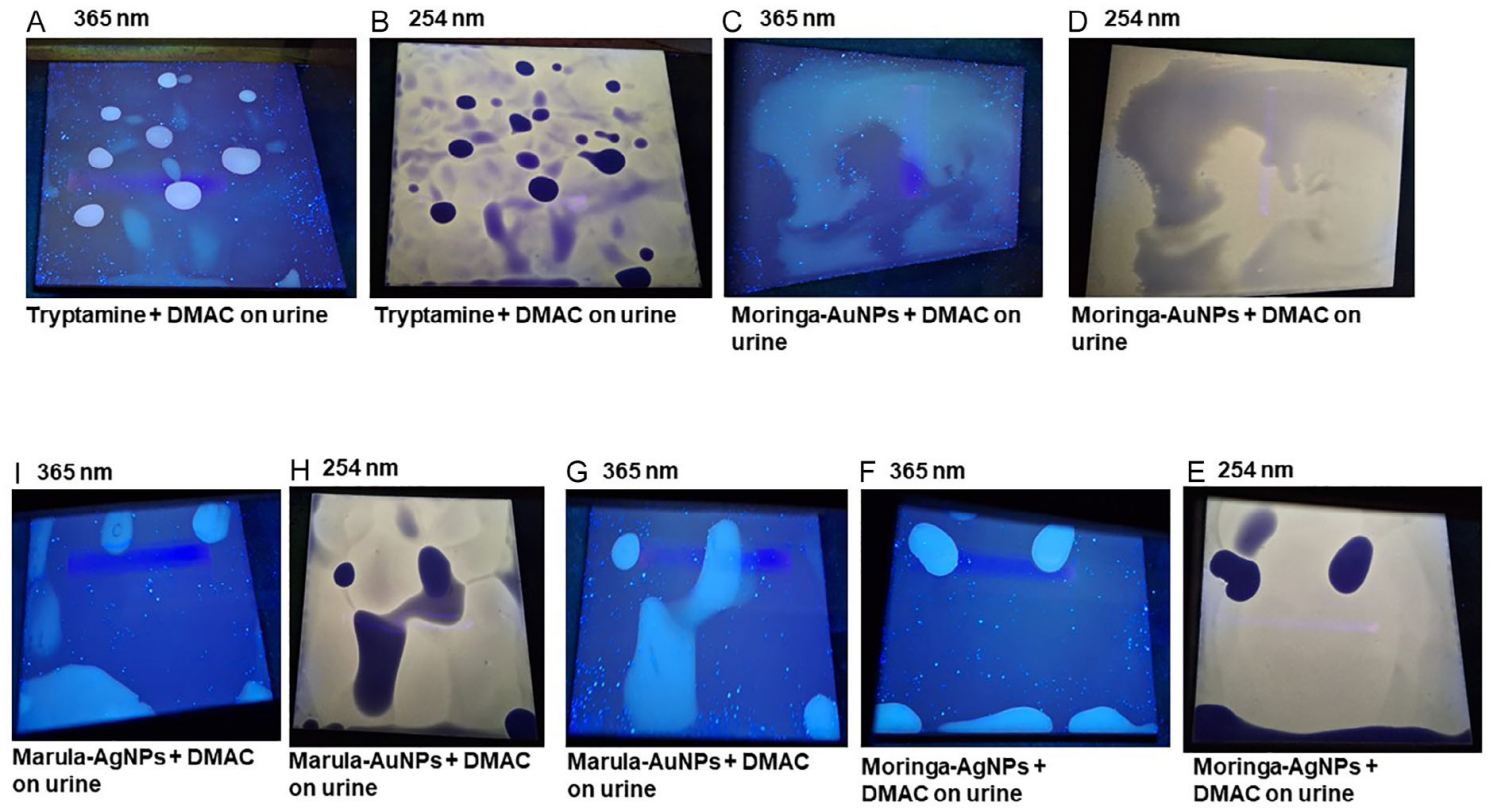
Analysis of urine under 254 and 365 nm using DMAC enhanced with tryptamine, silver, and gold nanoparticles.

The study also assessed the conventional reagent DMAC along with its modified variants, including DMAC‐based plant extracts, as illustrated in Figure 12. Although DMAC alone resulted in magenta color indicating the presence of urine, its fluorescence when exposed to UV light was minimal. Furthermore, since DMAC has the potential to degrade DNA, this negatively impacts its forensic use for the analysis of urine [10, 57, 58, 59]. To overcome this challenge, plant extracts were added to enhance the fluorescence of DMAC while maintaining the integrity of DNA in urine. The incorporation of *Sclerocarya birrea* extract led to notable visual and fluorescent enhancement: yellow fluorescence at 254 nm, blue fluorescence at 365 nm, and a light green color under ordinary lighting, as illustrated in Figure 12E–G. However, the DMAC‐*Moringa oleifera* sample appeared clear under regular light, resembling untreated urine. These results suggest that plant extracts can improve DMAC effectiveness without affecting DNA integrity. Additional fluorescence improvement was achieved by adding tryptamine and metal nanoparticles, as illustrated in Figure 13, which displayed strong fluorescence at 365 nm and fainter signals at 254 nm, confirming enhanced detection sensitivity under longer wavelength UV light.

**FIGURE 14:**
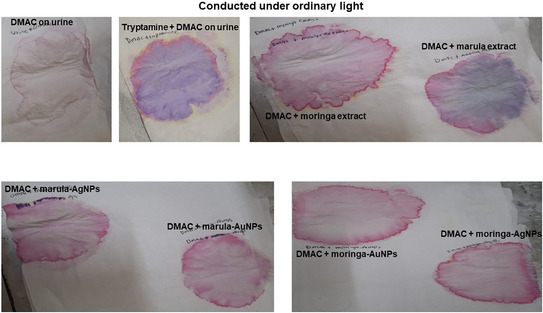
Analysis of urine on the paper towel under ordinary light using DMAC enhanced with *Marula* extract, *Moringa* extract, tryptamine, silver, and gold nanoparticles.

**FIGURE 15:**
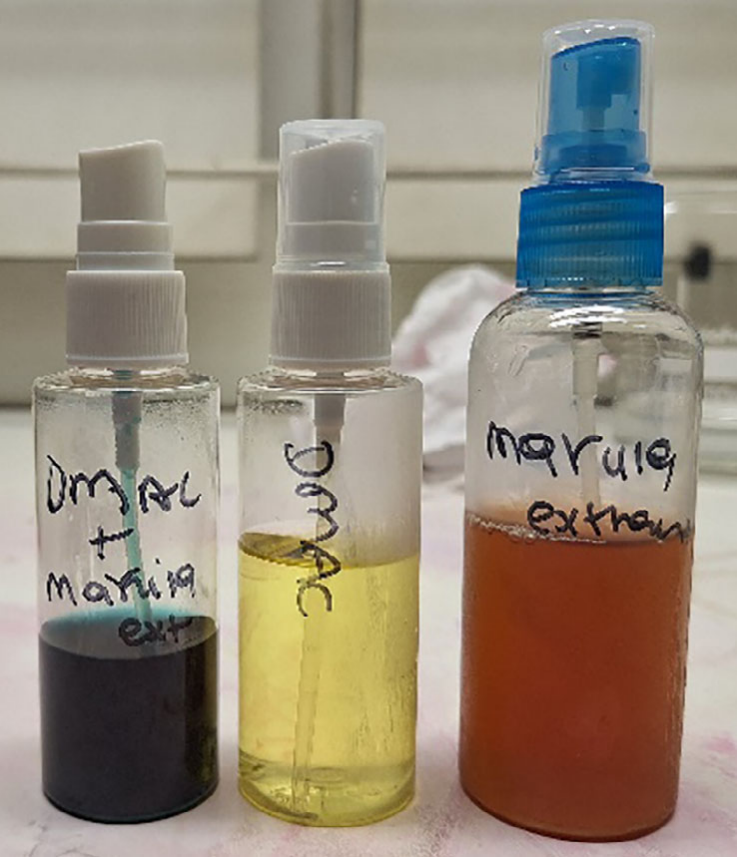
*Sclerocarya birrea* extract, DMAC, and a single product of DMAC and *Sclerocarya birrea*.

**SCHEME 1:**
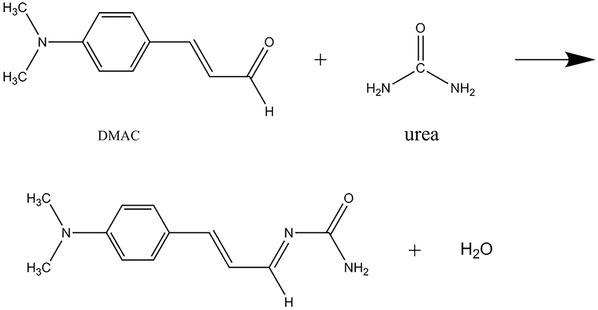
4‐Dimethylaminocinnamaldehyde (DMAC) + Urea → Schiff Base (which result in magenta color + water as aby product).

Human urine analysis utilizing paper towel substrates, as illustrated in Figure 14, revealed that DMAC alone produced a faint magenta color, while the incorporation of silver and gold nanoparticles and *Moringa extract* enhanced this to an intense magenta. Additionally, DMAC enhanced with tryptamine and *Sclerocarya birrea* extract resulted in a pink‐purple coloration. A new green product, resulting from mixing the DMAC reagent with *Sclerocarya birrea* extract, was further developed for detecting and analyzing urine on various surfaces, including tiles and paper towels, as illustrated in Figure 15. The color change is associated with the interactions between DMAC and phytochemicals, including polyphenols, flavonoids, and tannins found in *Sclerocarya birrea* (*Marula*) extract [60], along with potential complexation with trace metal ions like iron and copper found in the plant extract, which results in the formation of green chromophores through oxidation and coordination processes [61, 62, 63]. DMAC interacts with components of urine (such as urea and ammonia) to result in a magenta chromophore, which is subsequently enhanced by AgNPs and AuNPs through localized SPR, resulting in increased fluorescence at 365 nm and improved signal sensitivity as shown in Scheme 1 [37, 64]. Plant extracts provide strong intrinsic fluorescence due to phytochemicals (like flavonoids, polyphenols, and alkaloids), which increase additional fluorescent complexes with urine constituents and frequently dominate emissions at both 254 and 365 nm. In summary, urine detection is accomplished through the combined effects of DMAC chromophore formation, optical enhancement via nanoparticles, and fluorescent complexes between phytochemicals [65, 66, 67, 68, 69, 70] and urine, leading to enhanced visual under UV for forensic urine analysis. In summary, the research illustrates that incorporating plant extracts, tryptamine, AgNPs, and AuNPs significantly improves the sensitivity of urine analysis, especially at 365 nm, while reducing the DNA degradation associated with DMAC. This method offers a cost‐effective, nondestructive, and sustainable solution for forensic urine detection without affecting subsequent DNA analysis.

### 3.9 The Study of DNA Integrity and Purity

**FIGURE 16:**
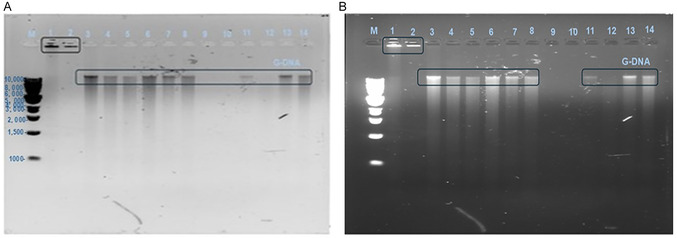
Analysis of DNA integrity using agarose gel electrophoresis following urine analysis with the reagents materials in (A) bright field and (B) dark‐field.

A DNA analysis study was carried out, with results displayed in Table 1 and Figure 16, to evaluate the integrity of DNA in urine samples before and after analysis with DMAC, plant extracts, AgNPs, and AuNPs for forensic DNA profiling. The urine samples that were not analyzed with DMAC, plant extracts, silver, and gold nanoparticles showed a DNA yield of 19,850 ng/µl (Test 8), while the application of DMAC notably decreased the DNA concentration to 3,600 ng/µl; A260/A280 = 1.204 (Test 9), indicating that the DNA was degraded by DMAC. In contrast, optimized DMAC with nanoparticles and plant extracts, as indicated in Tests 11, 13, and 14, maintained approximately 40% intact DNA, as verified by electrophoresis gels illustrated in Figure 16a,b, which display faint, but solid bands compared to the severe degradation observed in samples treated with DMAC alone. In comparison, saliva, as indicated in Table 1, exhibited significantly higher DNA concentrations in Test 1–7, with untreated saliva presenting 80,050 ng/µl in Test 1, demonstrating good purity (A260/A280 = 1.6–2.0) and clear high‐molecular‐weight bands resulting from a high presence of nucleated cells. These results correlate with electrophoresis gel results for urine DNA analysis reported by Zhi Shan et al. [71]. In contrast, urine DNA quantity was reduced and further affected by the presence of salts, urea, creatinine, and acids that naturally occur in urine [72], which contribute to degradation [73, 74, 75]. In summary, these findings indicate that while DMAC and higher concentrations of silver and gold nanoparticles can degrade DNA, the careful application of plant extracts and nanoparticles at lower concentrations can maintain the integrity of urine DNA, thus facilitating accurate forensic DNA profiling for identifying criminal suspects [72, 76, 77, 78] highlighted.

**TABLE 1:** Analysis of DNA concentration and purity in saliva and urine samples after analysis with plant extracts, 4‐dimethylaminocinnamaldehyde (DMAC), silver nanoparticles, and gold nanoparticles.

Samples	DNA concentration, ng/µl	Avg. absorbance at A260 nm (AU)	Avg. absorbance at A280 nm (AU)	A260/A280
1. Saliva	80, 050	1.630	0.919	1.799
2. Saliva + moringa extract	42, 302	0.871	0.464	1.929
3. Saliva + moringa‐AgNPs	36, 150	0.735	0.413	1.803
4. Saliva + moringa‐AuNPs	28, 200	0.567	0.323	1.762
5. Saliva + marula‐AgNPs	22, 750	0.724	0.411	1.832
6. Saliva + marula‐AuNPs	28, 550	0.602	0.364	1.715
7. Saliva + tryptamine	23, 750	0.585	0.421	1.763
8. Urine	19, 850	0.396	0.218	1.813
9. Urine + DMAC	3, 600	0.080	0.046	1.204
10. Urine + DMAC + marula‐AgNPs	10, 450	0.213	0.119	1.817
11. Urine + DMAC + marula‐AuNPs	11, 150	0.226	0.124	1.843
12. Urine + marula extract	7, 8500	0.213	0.373	1.743
13. Urine + DMAC + moringa‐AgNPs	9, 200	0.468	0.312	1.642
14. Urine + tryptamine	10, 750	0.313	0.416	1.747

## 4 Conclusion

This study demonstrates that the synthesis of silver nanoparticles (AgNPs) and gold nanoparticles (AuNPs) derived from *Marula* (*Sclerocarya birrea*) and *Moringa oleifera* using an easy, cost‐effective, and eco‐friendly technique improves urine analysis efficacy. The synthesized AgNPs and AuNPs demonstrated significant stability, with FTIR analysis indicating that phytochemicals from *Marula* and *Moringa* were vital in the reduction and stabilization of AgNPs and AuNPs. XRD analysis verified that the resultant nanoparticles had a crystalline structure, as demonstrated by the presence of the FCC phase. The study of particle size distribution and zeta potential indicated that the synthesized AgNPs and AuNPs exhibited diameters between 5 and 90 nm and possessed a negative charge, which is ascribed to their derivation from plant extracts. Moreover, these nanoparticles, along with *Marula* and *Moringa* stem extracts, were effectively utilized for urine analysis at crime scenes. A higher fluorescence signal was detected as a result of the interaction between urea and phytochemical substances, such as polyphenols, alkaloids, and flavonoids. 4‐Dimethylaminocinnamaldehyde (DMAC) alone could detect urine, but its fluorescence intensity appeared weak. The use of AgNPs and AuNPs derived from *Marula* and *Moringa* markedly improved the signal intensity. The results indicate that *Marula* and *Moringa* extracts, in conjunction with AgNPs and AuNPs, are effective, nontoxic alternatives for urine detection in forensic applications. The analysis of DNA integrity revealed that urine inherently has lower DNA concentrations compared to saliva, and extracting DNA from urine poses difficulties owing to its acidic environment and the presence of substances such as urea and creatinine that lead to degradation of DNA. The research demonstrated that 4‐dimethylaminocinnamaldehyde (DMAC) caused DNA degradation, whereas silver nanoparticles (AgNPs) and gold nanoparticles (AuNPs) showed protective properties against the damage induced by DMAC. While a higher concentration of nanoparticles and plant extracts was observed to damage DNA, carefully optimized concentrations successfully maintained DNA integrity.

## Author Contributions

**Leah**: conceptualization, methodology, visualization, and writing original draft preparation. **Eswaran Prabakaran**: methodology, editing, and interpretation of experimental data. **Pillay and Komane**: supervision, reviewing, editing, visualization, project administration, resources, and conceptualization.

## Funding

This study was supported by the National Research Foundation (SRUG2204082701).

## Conflicts of Interest

The authors declare no conflicts of interest.

## Data Availability

The data that support the findings of this study are available on request from the corresponding author. The data are not publicly available due to privacy or ethical restrictions.
